# Effects of short-term preoperative intranasal dexmedetomidine plus conventional treatment on delirium following cardiac surgery in patients with sleep disorders

**DOI:** 10.1186/s13741-024-00371-1

**Published:** 2024-03-09

**Authors:** Jun Fang, Jia Yang, Mingyu Zhai, Qiong Zhang, Min Zhang, Yanhu Xie

**Affiliations:** 1 Department of Anaesthesiology, The First Affiliated Hospital of USTC, Division of Life Sciences and Medicine, University of Science and Technology of China, Hefei, 230001 Anhui China; 2 Department of Cardiovascular Surgery, The First Affiliated Hospital of USTC, Division of Life Sciences and Medicine, University of Science and Technology of China, Hefei, 230001 Anhui China

**Keywords:** Dexmedetomidine, Preoperative intervention, Sleep disorders, Cognitive function

## Abstract

**Study objectives:**

To assess whether preoperative dexmedetomidine (DEX) nasal drips combined with conventional treatment could mitigate the occurrence of postoperative delirium (POD).

**Design:**

A prospective randomised controlled study.

**Setting:**

The cardiac surgery intensive care unit (CSICU) and patient hospitalisation ward at a university hospital.

**Participants:**

A total of 100 patients (aged ≥60 years) undergoing cardiac surgery at a university hospital between 7 January 2022, and 30 November 2022 met the eligibility criteria and were included in the study.

**Interventions:**

Patients with sleep disorders (Pittsburgh Sleep Quality Index ≥8) were divided into two groups: Group A (the placebo group, *n*=50), receiving a short-term preoperative placebo combined with conventional treatment and Group B (the DEX group, *n*=50), receiving short-term preoperative DEX combined with conventional treatment.

**Measurements and results:**

The Confusion Assessment Method for the ICU (CAM-ICU) was used for POD assessment in the CSICU, while the CAM was employed to assess delirium in the patient ward. Group B demonstrated a reduced incidence of POD compared to Group A (12% vs. 30%, odds ratio: 0.318, 95% confidence interval: 0.112–0.905, *p*=0.027).

**Conclusion:**

The combined treatment involving DEX demonstrated a decreased incidence of POD in elderly individuals with sleep disorders undergoing cardiac surgery compared to the placebo combination treatment.

**Trial registration:**

URL: www.chictr.org.cn with registration number ChiCTR 2100043968, registered on 06/03/2021.

## Introduction

Postoperative delirium (POD) is a common complication following cardiac surgery, with reported incidences ranging from 14.1% to 53.3% (Enomoto et al. 2023; Nakamura et al. 2023). Typically occurring 1–7 days after surgery, various factors contribute to cognitive function impairment, including patient age (Xu et al. 2022), cerebral embolism (Stump et al. 1996), cardiopulmonary bypass (Puskas et al. 2011), inflammatory response (Hudetz et al. 2011), preoperative cognitive ability (Greene NH et al.^2009^), opioid use (Fong et al. 2006), and inhaled anaesthesia (Eckenhoff et al. 2004). Research indicates that preoperative anxiety (Tully et al. 2010; Wada et al. 2019) and sleep disturbances (Todd et al. 2017; Wang et al. 2020) are also risk factors for POD. Dexmedetomidine (DEX), an α_2_-adrenergic receptor agonist, particularly the a_2A_ subtype mediating the hypnotic response in the locus coeruleus (Mizobe et al. 1996), has been applied in preoperative sedation (Zhang et al. 2021) and anxiolysis (Knaeps et al. 2023). Despite its known applications, there is limited literature on the short-term application of DEX nasal drip in patients with sleep disorders before surgery. Therefore, this study represents the initial endeavour to administer intranasal DEX to elderly patients with sleep disorders shortly before cardiac surgery. Eligible patients with sleep disorders scheduled for cardiac surgery were enrolled, receiving either DEX or a placebo nasal drip as an intervention before bedtime. The observation period in the special sleep treatment room (with first aid personnel and equipment) spanned from 9:30 pm to 6:30 am the following day. The primary objective of this study was to compare the effects of different intervention methods on the incidence of POD.

## Methods

### Patients

This study adhered to the principles outlined in the "Declaration of Helsinki" by the World Medical Congress and received approval from the Chinese Ethics Committee of Registering Clinical Trials under the ethics number ChiECRCT20210592. Additionally, it obtained approval and was documented with the Scientific Research Management of the First Hospital of the University of Science and Technology of China. The study was registered with the Chinese Clinical Trials Registry Centre at www.chictr.org.cn, with the registration number ChiCTR2100043968 (06/03/2021). All participants provided written informed consent before participating in the study.

The inclusion criteria were as follows: (1) patients who were aged between 60 and 75 years; (2) those classified as American Society of Anaesthesiologists grade III–IV; (3) those scheduled for cardiac surgery, such as off-pump coronary artery bypass grafting and valve or aortic root surgery, from 7 January 2022 to 30 November 2022; and (4) those with sleep disorders (defined as having a Pittsburgh Sleep Quality Index [PSQI] score of ≥8). The exclusion criteria were as follows: (1) patients who had a baseline Mini-mental Status Examination (MMSE) score of ≤21 points; (2) those who declined or were unable to participate in the study, communicate, or speak; (3) those with hearing disorders; (4) those with sleep apnoea syndrome; and (5) those who had an ejection fraction of ≤50% after admission. Criteria for withdrawal and termination of the study included patients who (1) had a preoperative hospital stay of <6 days; (2) experienced third-degree atrioventricular block; (3) were allergic to DEX; (4) were lost to follow-up; and (5) had data analysis failure.

### Study design

**Fig. 1:**
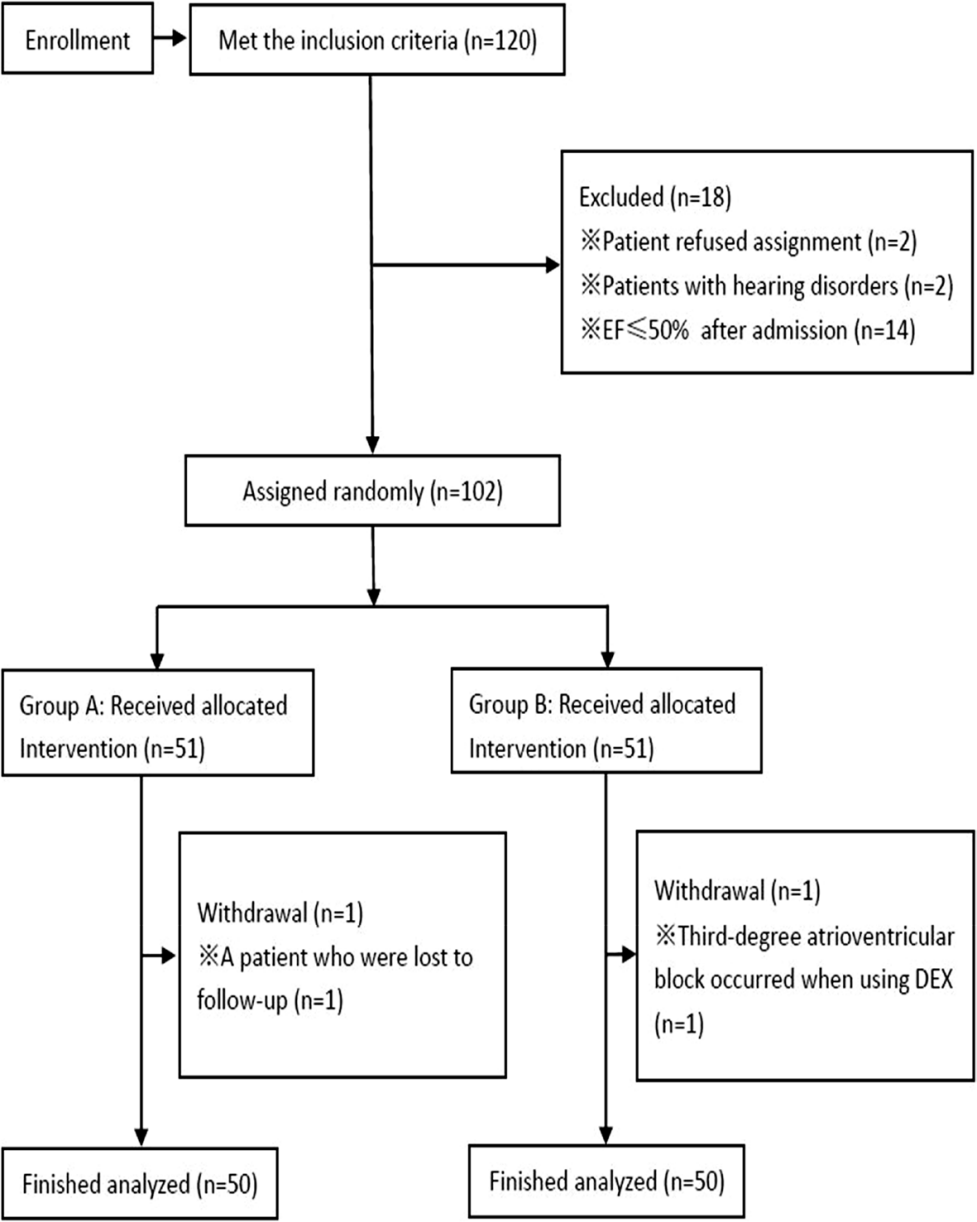
The technical flowchart of the clinical research on patients. *EF* ejection fraction, *DEX* dexmedetomidine

The flowchart representing the technical steps of the clinical research involving the patients is presented in Fig. 1.

### Grouping methods and double-blind design

The patients were randomly assigned in a 1:1 ratio using computer-generated numbers to form Groups A and B. The group allocations were sealed within sequentially numbered opaque envelopes, and these envelopes remained sealed until the patients provided informed consent. The patient, outcome assessor, and anaesthesiologist were blinded to the group assignment.

### Intervention methods

Patients received either DEX or saline nasal drip before bedtime for 6–8 days before the surgery, with a minimum of 1 day interval between each of the two or three interventions. The administration of the intranasal intervention was performed by either the sole anaesthetist or a clinically registered sedation doctor, directly supervised by the anaesthesiologist in the ward. The syringe used for administration comprised either 2 mL of normal saline or 0.3 µg/kg of DEX. Each syringe was used twice (1 mL for each nostril, administered 5 min apart). The patients were observed for 60 minutes, and if their Ramsay score was 5 points> Ramsay score ≥3 points (Kaur et al. 2016) and the bispectral index (BIS) was <70 (Kang et al. 2019) (indicating a sedation level comparable to natural sleep), the intervention was concluded. If not, an additional treatment dose was administered, and the patient was observed for another 60 min. No further treatment was administered after the third intervention, irrespective of the Ramsay score or the BIS. In terms of intranasal DEX dosage in adults, Yuen et al. (2007) reported DEX at doses of 1.0 and 1.5 µg/kg produced significant sedative effects compared to a placebo, causing reductions in blood pressure and heart rate (HR, *p*<0.05). Barends et al. (2020) used 0.5–2.0 μg/kg DEX nasal drips to achieve sedation. Even with the lowest dose (0.5 µg/kg DEX), the mean arterial pressure decreased by 30% for 5 min in 10% of patients. Our aim was to induce a state resembling “natural sleep” rather than general anaesthesia or deep sedation. The dose was titrated to improve the patient’s sleep while minimising the impact on circulation and avoiding respiratory depression. Standard anaesthesia procedures were implemented for all patients upon entering the operating room to mitigate the impact of the anaesthesia protocol on the study outcomes. Both groups received conventional treatment for sleep disorders, including the use of earplugs and maintaining a regular wake-sleep rhythm.

### Standard anaesthetic protocols

Upon the patient’s entry into the operating room for cardiac surgery, monitoring was initiated for the HR, invasive arterial pressure, central venous pressure, electrocardiogram, pulse oxygen saturation, and BIS. Anaesthesia induction involved administering 0.2–0.3 mg/kg etomidate, 0.5–0.9 mg/kg rocuronium, and 1.0–1.5 μg/kg sufentanil to all patients. Mask-assisted breathing ventilation was employed for 2 min, ensuring adequate muscle relaxation. The insertion of a tracheal tube with an appropriate inner diameter was performed using a visual laryngoscope (Insight iS2 Shenzhen Sultan Said Khan Medical Technology Co., Ltd.) when the BIS was maintained between 40 and 60. A fibreoptic bronchoscope was used to position the tube tip 2–3 cm distal from the tracheal carina. Mechanical ventilation parameters were set as follows: tidal volume of 6–10 mL/kg, an adjustable breathing rate to maintain partial pressure of end-tidal carbon dioxide of 35–45 mmHg, and an inhalation:exhalation ratio of 1:2. Inhalation of 1–2% of sevoflurane was administered, with intravenous anaesthetics maintained at 4–8 mg/(kg·h) of propofol, 0.1–0.3 mg/(kg·h) of cisatracurium, and a single supplement of 20–100 μg of sufentanil as appropriate.

### Definition of the outcome

The assessment of POD in the cardiac surgery intensive care unit (CSICU) was conducted every 12 h using the Confusion Assessment Method for the ICU (CAM-ICU) (Guenther et al. 2010), while delirium assessment at the ward employed the CAM. POD evaluation was performed 1-5 days postoperatively. A singular researcher administered the CAM-ICU or CAM measurement. Given the variations in specificity and sensitivity, the Diagnostic and Statistical Manual of Mental Disorders, fifth edition, was concurrently used for delirium screening. The secondary outcomes in this study encompassed postintervention anxiety, postintervention clinical insomnia, mechanical ventilation duration exceeding 12 h, occurrences of myocardial infarction, stroke, CSICU stay, hospital stay, and in-hospital mortality. Postintervention anxiety status was assessed a day before the surgery using the Self-rating Anxiety Scale (SAS). Postintervention sleep status (clinical insomnia) was evaluated for sleep quality approximately a day before the operation based on the insomnia severity index. Mechanical ventilation duration exceeding 12 h was reported as the percentage of time spent on ventilation from CSICU admission to ventilator withdrawal. The CSICU stay duration was recorded in hours, while the hospital stay was recorded in days. In-hospital mortality was defined as the number of patients who died from admission to discharge divided by the total number of patients in the study group.

### Calculation of sample size

In the pretrial phase, preoperative intervention with intranasal DEX drip resulted in a 10% incidence of POD, where one out of 10 patients exhibited this outcome. In contrast, recent studies (Enomoto et al. 2023; Nakamura et al. 2023) reported POD incidences ranging from 14.1% to 53.3%. Taking the median value of 33.7%, a two-tailed type I error rate of 5% and a test efficacy of 80% (α of 0.05 and β of 0.20) was assumed. Using Power Analysis Software 15, the sample size was calculated, indicating a requirement of 44 patients in each group. Considering a drop-out rate of approximately 10%, a sample size of 49 cases per group was suggested.

For continuous variables adhering to a normal distribution, the mean (standard deviation) was used for description. Non-normally distributed continuous variables were described as medians (interquartile ranges), while categorical variables were described as percentages. Between-group comparisons of continuous variables were compared using the independent sample t*-*test or Mann–Whitney U test. Categorical variables across different groups were compared using the chi-square test, and a two-sided *p*-value of <0.05 was considered statistically significant. Baseline variables influencing POD (*p*<0.05) were screened through a multivariate logistic regression model. All statistical analyses were performed using the Statistical Package for Social Science (SPSS) 22.0 (Chicago, IL, USA).

## Results

### Baseline data and intraoperative parameters

Baseline data in both groups included age; body mass index; sex; baseline PSQI score; preintervention anxiety; preintervention clinical insomnia; ejection fraction; presence of hypertension, diabetes, anaemia, and mild cognitive impairment (MCI); and the surgery type. Intraoperative data comprised variables such as operation time, anaesthesia time, sufentanil dosage, and propofol dosage. MCI was identified in patients with MMSE scores between 21 and 26. Serious adverse events included conditions such as hypotension, cardiac arrest, and heavy bleeding. Serious hypotension was defined as a systolic blood pressure of ≤80 mmHg occurring on two or more occasions, regardless of vasoactive drug use. Heavy bleeding was characterised by a loss of at least 50% of blood volume at the end of the surgery (Table 1).

**Table 1:** Comparison of baseline and intraoperative data between the two groups

**Characteristic**	**Group A (*n*=50)**	**Group B (*n*=50)**	***P* Value**
Age (y)	68.1(4.3)	66.9(3.4)	0.120
BMI (kg/m^2^)	23.8(3.4)	23.6 (2.5)	0.745
Gender (female), n (%)	22(44.0)	19 (38.0)	0.542
PSQI_0_ (median, IQR)	12.0 (10.8,14.0)	11.5(9.0,13.0)	0.071
Preoperative anxiety (SAS≥55), n (%)	15(30)	18(36)	0.523
Preintervention clinical insomnia (ISI≥15), n (%)	16 (32)	18 (36)	0.673
EF (%)	63.3 (6.3)	62.9 (7.3)	0.759
Smoking, n (%)	8(16.0)	7 (14.0)	0.779
Hypertension, n (%)	29(58.0)	27 (54.0)	0.687
Diabetes, n (%)	10(20.0)	10(20.0)	-
Anaemia, n (%)	10(20.0)	9 (18.0)	0.799
MCI, n (%)	8(16.0)	6 (12.0)	0.564
Type of procedure, n (%)			
Off-pump CABG, n (%)	14(28.0)	21(42.0)	0.142
Valve or aortic root surgery, n (%)	36(72.0)	29(58.0)	
Length of surgery (median, IQR, min)	292.5(250.0,332.5)	300.0(253.8,345.0)	0.684
Anaesthetic duration (median, IQR, min)	327.5(295.0, 397.0)	357.5(307.5, 401.3)	0.261
Sufentanil (median, IQR, µg)	361.0 (321.0, 395.0)	361.5 (341.0,407.5)	0.569
Propofol (median, IQR, mg)	1155.0(1012.0, 1337.0)	1200.0(1015.8,1463.0)	0.530
Serious event			
Serious hypotension, n (%)	5(10.0)	6(12.0)	0.749
Cardiac arrest, n (%)	0 (0)	1 (1.1)	>0.999*
Heavy bleeding, n (%)	0 (0)	0 (0)	-

Data are presented as the median (interquartile range) or n (%)

*BMI* Body mass index, *PSQI*_*0*_ baseline Pittsburgh sleep quality index, *IQR* interquartile range, *SAS* Self-Rating Anxiety Scale, *ISI* Insomnia severity index, *EF* Ejection fraction, *MCI* Mild cognitive impairment, *CABG* Coronary artery bypass grafting

^*^Fisher’s exact test

### Multivariate logistic regression analysis

To identify the factors that influence POD, the model was deemed to be significant when *p*<0.05. The results of the logistic regression analysis revealed that preoperative MCI, preoperative anxiety (SAS ≥55), and preoperative DEX intervention were the primary influencing factors (*p*<0.05, Table 2).

**Table 2:** Factors that may affect POD in the logistic regression analysis model

**Factors**	**Wald χ2**	***P* Value**
Age, years (≤70 or >70)	2.887	0.089
BMI,kg/m^2^ (≤25or >25)	0.237	0.626
Sex (Male or Female)	0.653	0.419
Preoperative anxiety (yes or no)	4.266	0.039
PSQI_0_ (<16 or ≥16)	3.613	0.057
Preintervention clinical insomnia (yes or no)	0.481	0.488
MCI (yes or no)	6.631	0.010
Surgery type (CABG or no-CABG)	0.000	0.985
EF (<55% or ≥55%)	0.129	0.719
Smoking (yes or no)	0.838	0.360
Hypertention (yes or no)	0.009	0.925
Diabetes (yes or no)	0.182	0.669
Anaemia (yes or no)	0.056	0.812
Surgery duration, min (<300 or ≥300)	1.274	0.259
Anesthaesia duration, min (<300 or ≥300)	1.008	0.315
Sufentanil, µg (<400 or ≥400)	0.386	0.534
Propofol, mg (<1250 or ≥1250)	0.547	0.460
Serious hypotension (yes or no)	2.968	0.085
Preoperative intervention (DEX or saline)	4.413	0.036

*BMI* Body mass index, *PSQI*_*0*_ Baseline Pittsburgh sleep quality index, *MCI* Mild cognitive impairment, *CABG* Coronary artery bypass grafting, *EF* Ejection fraction, *DEX* Dexmedetomidine

### Effects of the two different intervention methods on clinical outcomes

Table 3 illustrates the effects of the DEX and placebo interventions on various outcomes.

**Table 3:** Comparison of different clinical outcomes between Group A and Group B

**Overall outcomes**	**Group A (*n*=50)**	**Group B (*n*=50)**	***P* Value**
**Primary outcome**			
POD, n (%)	15 (30.0)	6 (12.0)	0.027
**Secondary outcome**			
Postintervention anxiety, n (%)	12(24)	4(8)	0.029
Postintervention clinical insomnia, n (%)	13(26)	5(10)	0.037
Mechanical ventilation duration>12 h, n (%)	41 (82.0)	40 (80.0)	0.799
Myocardial infarction, n (%)	2(4.0)	0 (0)	0.495
Stroke, n (%)	0 (0)	0 (0)	-
CSICU stay (median, IQR, h)	42.5 (21.8,63.3)	25.5 (19.0,67.5)	0.090
Hospital days (median, IQR, d)	23.5 (19.0,29.3)	24.0 (20.0,28.0)	0.997
In-hospital mortality, n (%)	0 (0)	0 (0)	-

Data are presented as the median (interquartile range) or n (%).*POD* postoperative delirium, *CSICU* Cardiac surgery intensive care unit, *IQR* interquartile range

Compared to Group A, Group B exhibited a significantly lower incidence of POD (12.0% vs. 30.0%, odds ratio [OR]: 0.318, 95% confidence interval [CI]: 0.112–0.905, *p*=0.027). Furthermore, postintervention anxiety decreased in Group B compared to Group A (8.0% vs. 24.0%; *p*=0.029). Notably, the incidence of postintervention clinical insomnia in Group B was significantly lower than that in Group A (10% vs. 26%; *p*=0.037).

### Incidence of anxiety and clinical insomnia in the two groups before and after the interventions

**Fig. 2:**
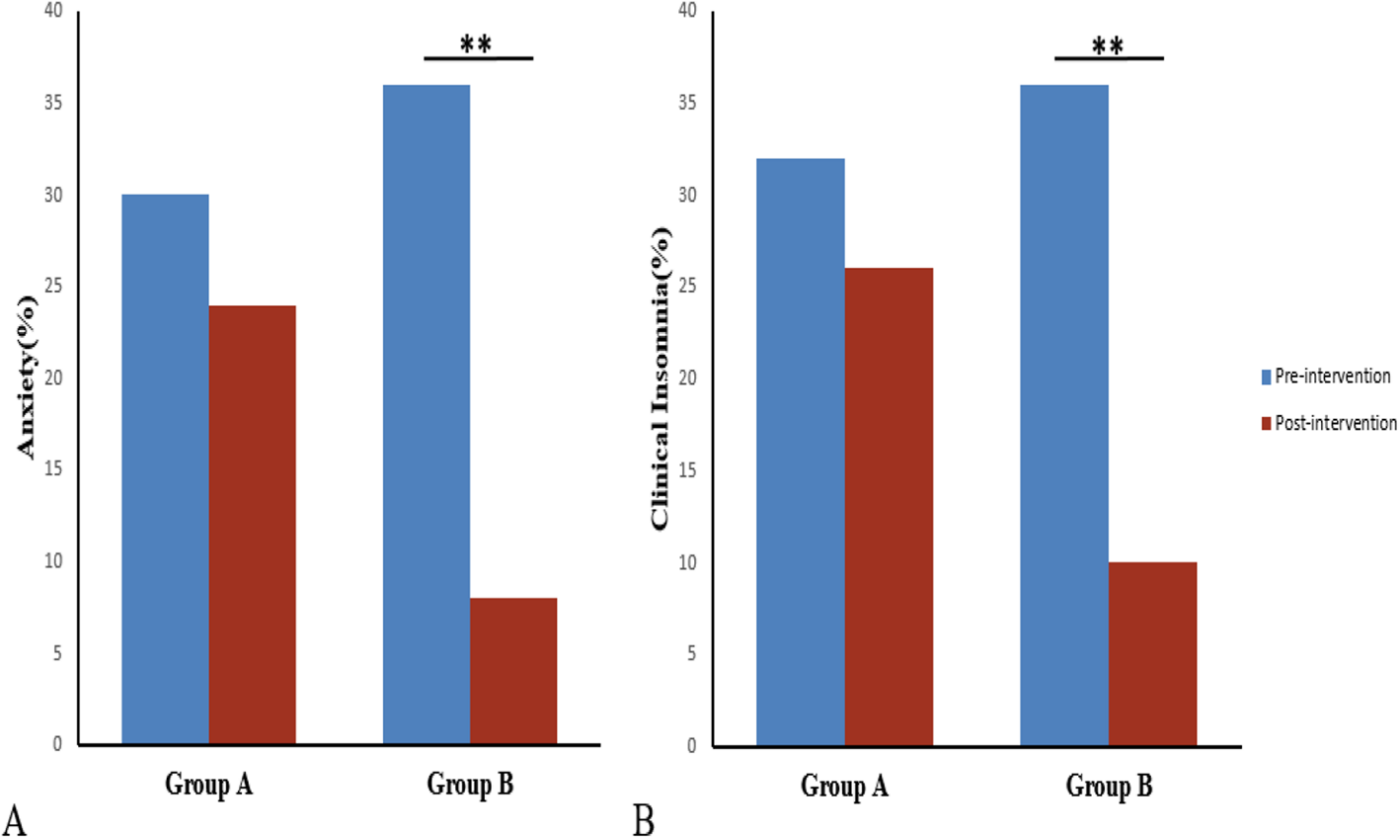
The incidence of pre-intervention and post-intervention anxiety (**A**) and clinical insomnia (**B**) in Group A and Group B. Each vertical bar is represented as n (%). ** p <0.01

After the intervention, there was a decrease in the incidence of anxiety in Group B (36% vs. 8%; *p*=0.001), whereas Group A did not exhibit a significant reduction (30% vs. 24%; *p*=0.499, Fig. 2A). This suggests that the combination of DEX with conventional treatment methods effectively decreased the occurrence of anxiety. In terms of sleep quality assessment, Group B demonstrated a reduction in the incidence of clinical insomnia (36% vs. 10%; *p*=0.002), while Group A did not show a significant decrease (32% vs. 26%; *p*=0.509, Fig. 2B).

## Discussion

Previous studies (Yuen et al. 2007; Iirola et al. 2011) have demonstrated that intranasal DEX injection is an effective and well-tolerated option for mild sedation in patients, *indicating* an absolute bioavailability of approximately 65%. Additionally, intranasal DEX has been observed to induce sedation in elderly patients (age ≥65 years), although caution is advised regarding the occurrence of hypotension in this population (Barends et al. 2020). In the current study, it is postulated that DEX might emulate a state similar to “natural sleep” by modulating the endogenous nonrapid eye movement sleep pathway (Akeju et al. 2018).

In a prospective cohort study, Wada et al. (2019) used multivariate logistic regression to analyse 91 patients with POD, revealing that preoperative anxiety effectively predicted delirium episodes (OR: 4.370, 95% CI: 1.051–18.178, *p*=0.043). Therefore, compared to the placebo group, the incidence of POD in the DEX group in our study was significantly lower (12.0% vs. 30.0%, OR: 0.318, 95% CI: 0.112–0.905, *p*=0.027), potentially attributed to the lower incidence of postintervention anxiety in the DEX group compared to the placebo group (8% vs. 24%, *p*=0.029). The precise mechanism by which anxiety contributes to delirium remains unclear. It is hypothesised that the connection between anxiety and delirium might be associated with shared central neuroinflammatory pathways implicated in their development (Inouye et al. 2014; Vasunilashorm et al. 2015; Furtado and Katzman 2015; O’Donovan et al. 2010). Additionally, the lower incidence of POD in the DEX group compared to the placebo group (*p*=0.027) could be attributed to the reduced incidence of postintervention clinical insomnia in the DEX combination treatment group compared to the placebo combination treatment group (10% vs. 26%, *p*=0.037). A systematic evaluation and meta-analysis of 1,238 citations revealed a significant association between sleep disturbance and POD (OR: 5.24, 95% CI: 3.61–7.60, *p*<0.001) (Fadayomi et al. 2018). The potential pathophysiological links between sleep disorders and delirium encompass neurotransmitter imbalances, deficiencies of certain substances such as vitamin D, and altered melatonin metabolism, resulting in diminished neuroprotection (Dessap et al. 2015; Campbell et al. 2019; Hshieh et al. 2008; Velayati et al. 2020).

Preoperative DEX intervention, preoperative anxiety, and preoperative MCI emerged as the primary influencing factors for POD (*p*<0.05, Table 2). These findings align with prior reports indicating that preoperative anxiety (Tully et al. 2010; Wada et al. 2019) and preoperative MCI (Wang et al. 2022) contribute to an increased POD incidence. Although the proportion of mechanical ventilation time exceeding 12 h in Group A was higher than that in Group B (82% vs. 80%), this difference was not statistically significant (*p*=0.799). Other indicators, such as myocardial infarction, stroke, the length of hospital stay, and in-hospital mortality, did not exhibit significant differences between the two groups.

Our study has several limitations. First, patients in the two intervention groups received additional conventional treatments for sleep disturbances. The non-uniformity in the effects of these conventional treatments on sleep disturbances might introduce a degree of variability that could affect the results. Second, POD was assessed in a 24-h postoperative evaluation period, which might be affected by the residual effects of intraoperative anaesthetics and medications administered during the CSICU stay. Third, the successful implementation of this study required a specific location, specialised monitoring equipment, and trained researchers. Therefore, the application of this clinical technique is limited to medical institutions with appropriate conditions, and widespread adoption in primary care settings might pose challenges.

## Conclusion

In elderly patients with sleep disorders undergoing cardiac surgery, the use of preoperative intranasal DEX combined with conventional treatment could reduce the incidence of POD compared to the combination of a placebo with conventional treatment. Therefore, for eligible elderly individuals, the suggestion is to consider administering intranasal DEX combined with conventional treatment before surgery. As an avenue for future exploration, there is a need for cost-effective medications with fewer side effects to serve as alternatives to DEX to reduce the POD incidence among patients with sleep disorders, with a particular focus on their viability as nasal drops.

## Data Availability

The data supporting this research can be obtained from the corresponding author upon reasonable request.

## Abbreviations

BIS: Bispectral index
BMI: Body mass index
CABG: Coronary artery bypass grafting
CAM-ICU: Confusion Assessment Method for the Intensive Care Unit
CI: Confidence interval
CSICU: Cardiac surgery intensive care unit
DEX: Dexmedetomidine
EF: Ejection fraction
HR: Heart rate
IQR: Interquartile range
ISI: Insomnia severity index
MCI: Mild cognitive impairment
MMSE: Mini-mental Status Examination
OR: Odds ratio
POD: Postoperative delirium
PSQI_0_: Baseline Pittsburgh Sleep Quality Index
SAS: Self-rating Anxiety Scale
